# All-in-One Pediatric Parenteral Nutrition Admixtures with an Extended Shelf Life—Insight in Correlations between Composition and Physicochemical Parameters

**DOI:** 10.3390/pharmaceutics13071017

**Published:** 2021-07-02

**Authors:** Aleksandra Gostyńska, Joanna Starkowska, Paulina Sobierajska, Anna Jelińska, Maciej Stawny

**Affiliations:** Department of Pharmaceutical Chemistry, Poznan University of Medical Sciences, Grunwaldzka 6, 60-780 Poznań, Poland; agostynska@ump.edu.pl (A.G.); asia.starskowska@gmail.com (J.S.); paulinaso96@gmail.com (P.S.); ajelinsk@ump.edu.pl (A.J.)

**Keywords:** parenteral nutrition, parenteral amino acid solution, lipid emulsion, stability

## Abstract

The administration of three-in-one parenteral nutrition (PN) admixtures to pediatric patients requires special consideration, specifically concerning quality and physicochemical stability. The introduction of a new parenteral amino acid solution into the market prompted us to evaluate Aminoplasmal Paed-based PN admixtures’ stability. The study aimed to determine the physicochemical parameters of the chosen variations of PN admixtures and search for a correlation between its composition and those parameters. One hundred and sixty-eight variations of PN admixtures intended for patients weighing from 10 to 25 kg and aged from 1 to 12 years and differing in the quantitative composition of electrolytes were selected for the study. The samples were prepared using each of the four intravenous lipid emulsions dedicated to pediatric patients: Intralipid 20%, Clinoleic 20%, Lipidem 20%, and Smoflipid 20%. The stability of the PN admixtures was assessed by visual inspection and determination of pH, osmolality, zeta potential, and hydrodynamic mean droplet diameter (MDD) immediately upon preparation and after seven days of storage at the temperature of 5 ± 1 °C with light protection. Pearson’s correlation was used to quantify the relationships between selected ingredients of the PN admixtures and the physicochemical parameters. The PN admixtures were characterized by pH ranging from 5.91 to 7.04, osmolality ranging from 1238 to 1678 mOsm/kg, and zeta potential ranging from −41.3 to −2.16 mV. The changes in pH and osmolality after seven days of storage did not exceed 0.2 and 4.4%, respectively. The homogeneity of the PN admixtures was confirmed by determining the polydispersity index, which ranged from 0.06 to 0.2. The MDD of the studied formulas ranged from 235 to 395 nm and from 233 to 365 nm immediately upon preparation and after the storage period, respectively. Correlations between selected components of the PN admixtures and some physicochemical parameters were found. All Aminoplasmal Paed 10%-based PN admixtures were characterized by appropriate physicochemical quality to be administered via the central veins, both immediately upon preparation and after seven days of storage at the temperature of 5 ± 1 °C with light protection. The applied electrolyte concentrations ranges and types of lipid emulsions in the selected macronutrient quantitative compositions allowed the PN admixtures to remain stable for seven days within the specified limits.

## 1. Introduction

The provision of parenteral nutrition (PN) in pediatric patients poses many unique considerations and challenges. The composition of the PN admixture used in pediatric patients is tailored to meet the daily nutrients and energy demand to ensure the maintenance e of proper nutritional status and allow appropriate growth and development [1]. Pediatric patients need different amino acid content and composition to the adult population due to hepatic immaturity and undeveloped enzymatic systems. This applies especially to preterm infants and neonates. Pediatric amino acid solutions, as compared with standard formulations originally designed for adults, present unique amino acid compositions that result in a plasma amino acid pattern resembling the plasma amino acid patterns of normally growing, breastfed infants and children [2]. The provision of such solutions aims to ensure the adequate administration of essential and conditionally essential amino acids, such as cysteine, glutamine, glycine, histidine, taurine, and tyrosine [3]. 

The provision of a lipid emulsion in pediatric patients is necessary, as it is the source of energy and essential fatty acids. Pharmaceutical preparations of lipid emulsions differ in content and sources of fatty acids. The type of lipid emulsion used in a PN admixture may affect a patient’s clinical outcomes, especially in the case of long-term PN administration [4,5]. A supply of a lipid emulsion with an inappropriate composition may lead to a deficiency of essential fatty acids or PN-related complications. For example, omega-3 fatty acids such as docosahexaenoic acid (DHA) and eicosapentaenoic acid (EPA) are regarded as conditionally essential for children and neonates, as they present a critical role in the development of the brain and retina of the eye [5]. Although the pathogenesis of the intestinal failure-associated liver disease is multifactorial, a growing body of evidence suggests that the dose of soybean oil-based lipid emulsion and the phytosterols contained therein are involved in the development of liver dysfunction. Thus, the administration of PN admixtures containing only soybean oil may lead to its development [4]. Lipid emulsions containing other sources of fatty acids, for example, omega-3 fatty acids from fish oil, may be potentially beneficial compared with solely soybean oil-based lipid emulsion as they characterized by lower omega-6 and higher omega-3 polyunsaturated fatty acids (PUFAs) concentrations, high concentrations of α-tocopherol, and reduced phytosterol content. Additionally, EPA and DHA exhibit significant clinical benefits by suppressing inflammatory mediators and activating anti-inflammatory pathways [4,5,6]. For many years, due to insufficient evidence, the use of lipid emulsions other than those based solely on soybean oil in pediatric patients was avoided. This situation has changed in recent years as new data confirming the benefits and safety of the use of lipid emulsions containing medium-chain triglycerides, olive oil, and fish oil as a source of fatty acids in this population were published [7,8]. However, despite theoretical data and some evidence showing improvement in children’s outcomes when using lipid emulsion, which is a mixture of different oils in comparison with solely soybean oil-based types, there is still a lack of consensus and official recommendations on which type of lipid emulsion is best for children of different ages. 

The stability of pediatric PN admixtures is a frequent subject of analysis and research [9,10,11,12,13]. These studies result from the need to ensure the safety of the intravenous administration of PN admixtures in this specific and sensitive group of patients. The PN admixture is a complex drug, the administration of which is associated with a risk of serious medical complications. This is mainly due to the presence of lipid emulsion, which is characterized by relatively large droplets and a lack of thermodynamic stability. The administration of lipid droplets exceeding the diameter of 500 nm may lead to catheter occlusion and liver capillaries’ embolization [14]. The stability of the lipid emulsion can be affected by the components present in the PN admixture, especially ions [15]. For this reason, in the pediatric population, PN therapy was conducted mainly using two-in-one PN admixtures containing amino acid solution, glucose, electrolytes, and water-soluble vitamins, and lipid emulsion was administered separately. Such an administration regime aims to avoid the interaction between lipid emulsion and components of the water phase of the PN admixture. Although three-in-one PN admixtures containing all nutrients mixed in one container are not necessarily therapeutically advantageous in the neonatal population, certain benefits may exist that exceed the risk in the elderly pediatric population. The advantages resulting from such administration are the potential to reduce the risk of contamination due to a decreasing number of manipulations, and administration facilitation, which is particularly important in the home care setting [16]. Nevertheless, three-in-one PN admixtures cannot be administered without the data confirming their physicochemical stability. These studies should consider the concentrations of individual macro and micro components in PN admixtures and take into account other seemingly less important details such as the type of pharmaceutical preparation and its detailed qualitative and quantitative composition. Stable PN admixtures may lose their original quality due to replacing the amino acid solution with another or using a different lipid emulsion. This change may result from the qualitative and quantitative composition, including pharmaceutical excipients in a given preparation. The composition of the source materials affects the physicochemical properties, such as pH, osmolality, and in the case of intravenous lipid emulsion, mean droplet diameter (MDD), and zeta potential. Further, all these features affect the final product (PN admixtures). For this reason, before the application of a PN admixture containing a new medicinal product (used as source material) in clinical practice, detailed physicochemical analysis of the individual components should be performed in a wide range of concentrations. Our study aimed to determine the stability of one hundred and sixty-eight variations of pediatric PN admixtures intended for pediatric patients, containing a recently approved amino acids solution, four different lipid emulsions, and different electrolyte concentrations, and searching for the correlation between the composition of the PN admixtures and their physicochemical parameters. The lipid emulsions selected for the study differed in oil composition, physicochemical parameters, and pharmacological activities. Therefore, confirmation of the stability of the PN admixtures containing Aminoplasmal Paed 10% and different types of lipid emulsions may be of great interest to clinicians.

## 2. Materials and Methods

### 2.1. Preparation of PN Admixtures 

**Figure 1:**
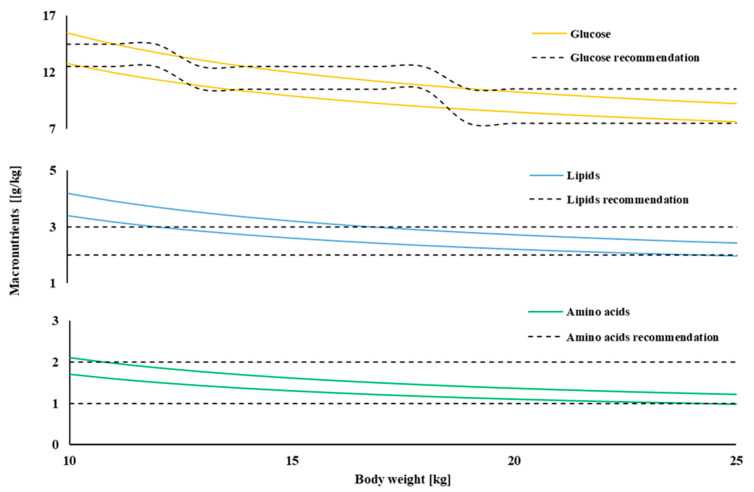
The macronutrients doses ranges in the function of patients’ bodyweight in relation to ESPEN/ESPGHAN recommendation.

One hundred and sixty-eight variations of PN admixtures based on Aminoplasmal Paed 10% (B. Braun Melsungen AG, Melsungen, Germany) were prepared by hospital pharmacists under aseptic conditions in accordance with our national pharmaceutical standards. The test solutions were stored in 200 mL ethylene-vinyl acetate bags (Nutrimix 200 mL, B. Braun, Melsungen AG, Melsungen, Germany). PN admixtures selected for the study differed in the content of electrolytes and the type of lipid emulsion (see Table 1). The composition of the PN admixtures was developed on the basis of the ESPGHAN guidelines for patients weighing from 10 to 25 kg and aged from 1 to 12 years. Taking into account the fact that the need for fluids increases with increasing body weight, PN admixtures with the same concentration range may be appropriate for children of different weights. Therefore, the concentrations of amino acids and glucose in individual PN admixtures did not vary significantly. We calculated the fluid requirements using standard formulas with the adjustments used by De Cloet et al. [17], i.e., 100 mL/kg/day for the first 10 kg body weight and 30 mL/kg/day for the remaining 1–15 kg. In the presented research model, the bodyweight of pediatric patients in a given age group was calculated as the mean body weight for the 10th percentile for boys and girls. In Figure 1 the macronutrients’ dose ranges in the function of patients’ body weight in relation to ESPEN/ESPGHAN recommendation are presented. Four different types of lipid emulsion were used: Intralipid 20% consisted solely of soybean oil; Clinoleic 20% consisted of soybean oil and olive oil; Lipidem 20% consisted of soybean oil and coconut oil enriched with omega-3 fatty acid triglycerides; and Smoflipid 20% consisted of soybean oil, olive oil, coconut oil and fish oil rich in omega-3 fatty acid. Detailed composition of PN admixtures and summarized composition expressed as concentration ranges of the particular compounds are presented in Supplementary Materials Table S1 and Table 1, respectively.

### 2.2. Stability Assessment

The stability of PN admixtures was assessed by visual inspection and determination of pH, osmolality, MDD of lipid emulsion, and zeta potential. The visual inspection and physicochemical parameters determination were performed immediately upon preparation (t = 0 h) and after seven days of storage at the temperature of 5 ± 1 °C with light protection. Each sample was measured in triplicate, and the results were expressed as average ± standard deviation. All measuring apparatuses were calibrated before use according to the manufacturer’s instructions. As a positive control, we used ready-to-use PN admixtures (Lipoflex special, B. Braun Melsungen AG, Melsungen, Germany) immediately after activation. Lipoflex special subjected to stress factors (exposure to 150 °C for 30 min or addition of 0.1 mol/L HCl at 1:1 volume ratio) were used as a negative control. Both the positive and the negative controls were performed in triplicate.

#### 2.2.1. Visual Inspection

In accordance with the European Pharmacopoeia [18], all PN admixtures were visually assessed for the presence of visible particles and color change. Visual inspection was performed against a black-and-white contrast background under the appropriate intensity of illumination by two observers. For visual inspection, samples of PN admixtures were transferred into 10 mL tubes with an internal diameter of 1 cm. In the case of any doubts about PN admixture instability manifested with precipitation, the microscopic inspection was performed.

#### 2.2.2. pH Evaluation

The pH was measured at room temperature, using Mettler Toledo Seven Compact pH/ion S220^®^ pH-meter (Mettler Toledo, Columbus, OH, USA). 

#### 2.2.3. Osmolality Determination

The osmolality was measured at room temperature, using an 800 CLG TridentMed^®^ osmometer (TridentMed s.c., Warsaw, Poland). We injected 100 μL of PN admixture into the Eppendorf tube, and the tube was placed in a cooling chamber. 

#### 2.2.4. MDD of Lipid Emulsion and Zeta Potential Determination

The MDD of lipid emulsion and zeta potential of PN admixtures were measured at 25 °C, using a Zetasizer Nano ZS (Malvern Instruments, Malvern, UK) by dynamic light scattering (DLS) and laser Doppler velocimetry, respectively. The droplet size and the zeta potential determination were performed according to the methodology described in our previous work [19]. The samples’ homogeneity was determined and expressed as a polydispersity index (PDI).

### 2.3. Acceptance Criteria

The following criteria must be met immediately upon preparation and after the storage to consider PN admixture as stable and presenting satisfactory quality. Following the European pharmacopeia requirements for intravenous lipid emulsions, PN admixtures must be practically free from visible particles, and no precipitation can be detected by either observer upon visual inspection [18]. The size of lipid droplets expressed as intensity-weighted MDD cannot exceed the pharmacopeial limit of 500 nm. This criterion was set for the US Pharmacopeia method I for the determination of the MDD of lipid injectable emulsions [20]. We established the acceptance criteria for the remaining parameters based on the work of other researchers and our own experiences [19,21,22,23,24,25,26,27]. The changes in measured parameters over time cannot exceed the values of ±0.2 and ±5% for the pH and osmolality, respectively. The zeta potential cannot take a positive value. The polydispersity index (PDI) has to be ≤0.7 since values > 0.7 indicate the lack of homogeneity of the oil-in-water system. 

### 2.4. Statistical Analysis

The data were analyzed using Statistica 12 software (StatSoft). Pearson’s correlation was used to quantify relationships between selected ingredients of PN admixtures and the physicochemical parameters. Due to slight differences in the content of amino acids, glucose, and lipids, only the following qualitative factors were used for the correlation analysis: the type of lipid emulsion, the content of sodium, potassium, magnesium, calcium, the sum of monovalent ions, and the sum of divalent ions.

## 3. Results

All variations of PN admixtures were homogeneous oil-in-water emulsions free from visible particles and characterized by pH ranging from 5.91 to 7.04 and osmolality ranging from 1238 to 1678 mOsm/kg. Pearson analysis showed a high correlation coefficient between the concentration of phosphate ions and the pH value (0.9195). The highest pH values were observed for formulas containing high content of glycerophosphate sodium, a source of phosphates in PN admixtures. High correlation coefficients were observed between sodium concentration (0.8610), potassium concentration (0.8783), the sum of those ion concentrations (0.8806) and osmolality. After seven days of storage, no significant changes in pH were observed, with a maximum difference of 0.02. The changes in osmolality after seven days did not exceed 4.4%. The PN admixtures showed negative zeta potential and rage from −41.3 to −2.16 mV. The lowest absolute values of zeta potential were observed for those formulas that contained the highest concentration of divalent ions (calcium and magnesium) and were highest for samples without those electrolytes. The zeta potential values correlated with calcium concentration (0.6639) and with the sum of magnesium and calcium ions (0.7314). The storage of PN admixtures affects the zeta potential values, which differed from the results obtained upon preparation. However, in none of the PN admixtures did the zeta potential undergo charge conversion.

**Figure 2:**
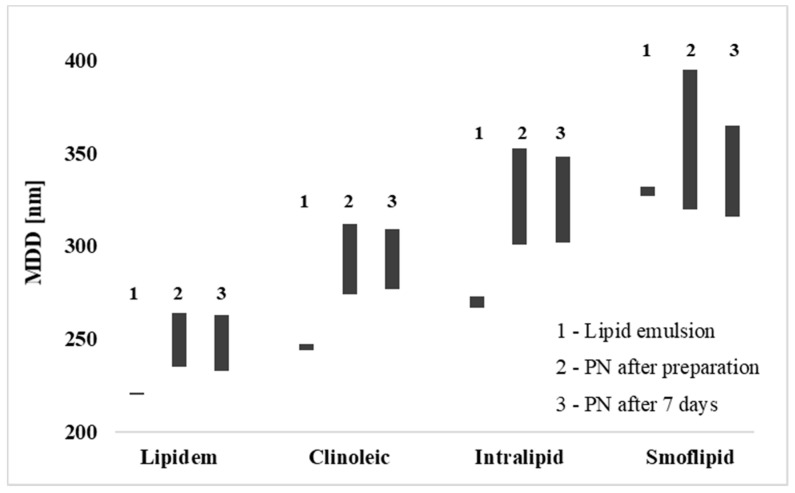
Mean droplet diameter range for studied PN admixtures and used lipid emulsions.

The homogeneity of the PN admixtures was confirmed by determining the PDI, which ranged from 0.06 to 0.2 and did not undergo significant changes after the storage period. The MDD of the studied formulas ranged from 235 to 395 nm and from 233 to 365 nm immediately upon preparation and after seven days of storage, respectively. The MDD of the studied batches of lipid emulsions amounted to: 221 ± 1 nm for Lipidem, 247 ± 3 nm for Clinoleic, 272 ± 6 nm for Intralipid 20%, and 331 ± 5 nm for Smoflipid. The MDD values obtained immediately upon preparation correlated with the lipid emulsion used (Figure 2). The smallest droplets were observed in PN admixtures containing Lipidem 20% as sources of lipids compared to PN admixtures containing Smoflipid 20%, where the droplets were the biggest. Both preparations are enriched with omega-3 fatty acids but differ in the content of other sources of triglycerides. Despite differences in droplet size, the pharmacopeial criterion of MDD < 500 nm for intravenous lipid emulsion was met for all studied formulas (Figure 2).

**Figure 3:**
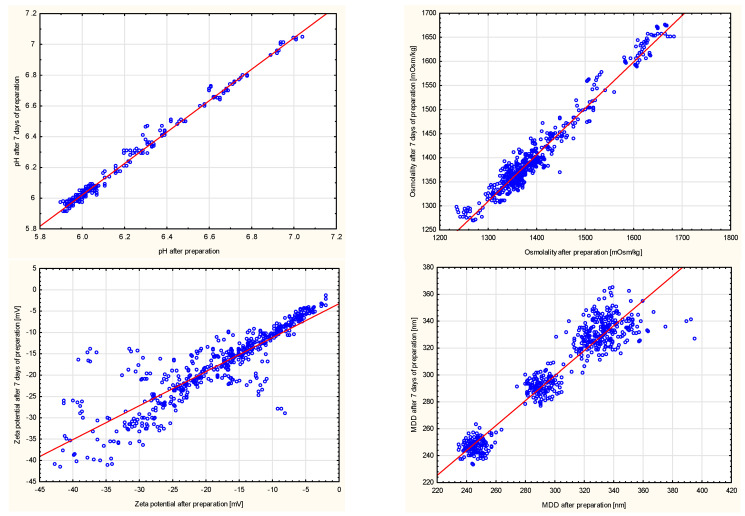
Correlation of pH, osmolality, zeta potential, and MDD immediately preparation and after seven days of storage.

**Figure 4:**
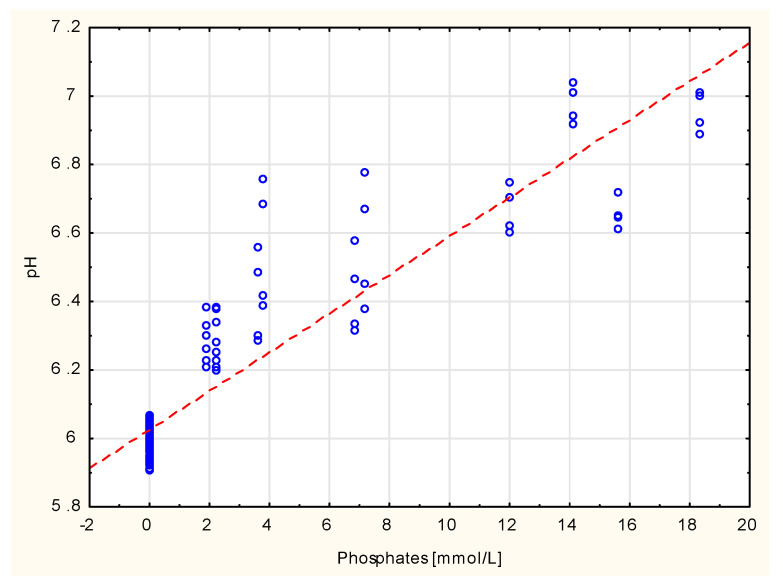

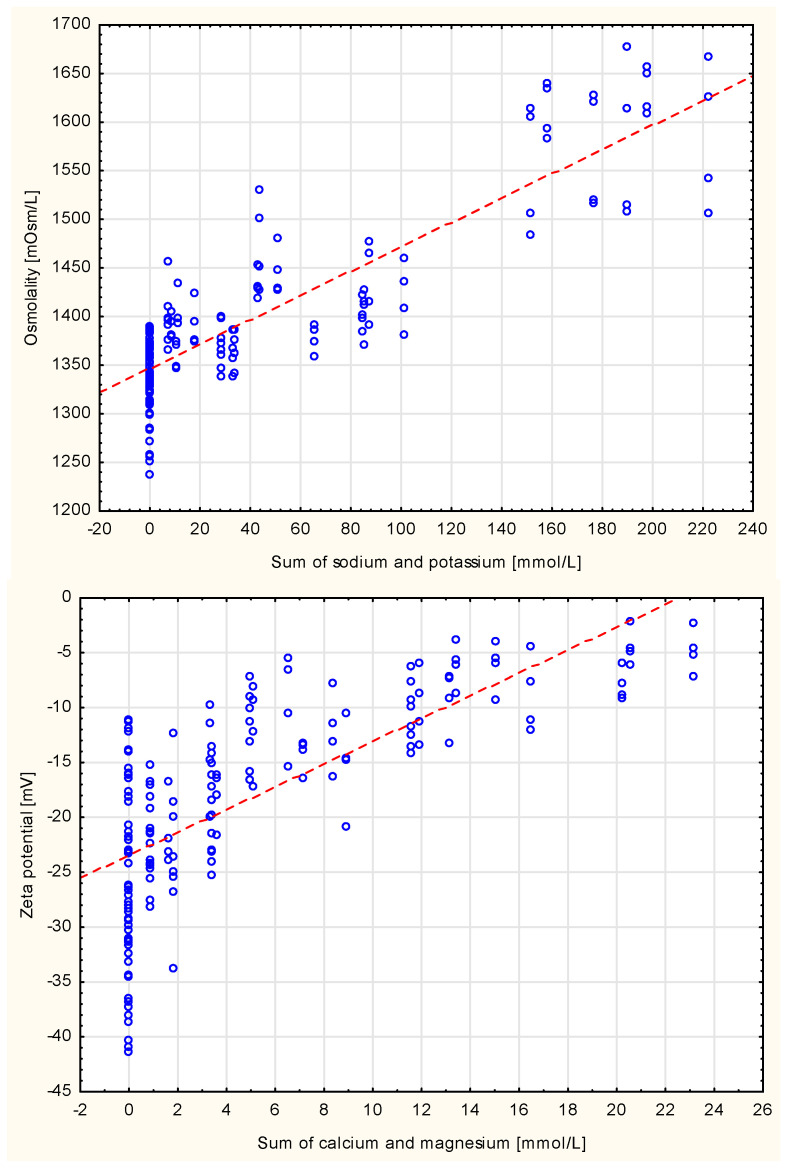
Correlation between selected physicochemical properties and ingredients of PN admixture.

Pearson’s correlation was also conducted separately for the results obtained for each type of lipid emulsion used. The statistical analysis carried out in such a manner showed that, regardless of the lipid emulsion used, there is a similar correlation for individual qualitative factors and selected physicochemical parameters. In the case of MDD, however, no statistically significant correlation coefficient between analyzed qualitative factors and this parameter was found, proving that the main factor affecting MDD is the type of lipid emulsion. The results described above are presented in detail in Supplementary Table S2 and graphically in Figure 3 and Figure 4.

## 4. Discussion

The physicochemical parameters of one hundred and sixty-eight different PN admixtures were determined and compared with their acceptance criteria in relation to results obtained for PN admixtures immediately upon preparation and the differences between the results obtained on the first day and after seven days of storage. In order to investigate the effect of the type of lipid emulsion on the physicochemical parameters of the three-in-one PN admixtures, each formula differing in the content of individual components (amino acids, glucose, lipid emulsion, electrolytes) was prepared in four variants depending on the lipid emulsion used: Intralipid 20%, Lipidem 20%, Smoflipid 20%, Clinoleic 20%. PN admixtures used as intravenous pharmaceutical preparations must meet the criteria established for intravenous dosage forms, such as sterility, non-pyrogenicity, and no particulate contamination [18]. Moreover, they have to be physicochemically and microbiologically stable for a minimum of 24 h at room temperature, i.e., from preparation to the end of the infusion [15]. Due to the impossibility of sterilization of the PN admixture, its preparation takes place in validated aseptic conditions from sterile medicinal products. Although many analytical methods are used to assess the quality of intravenous lipid emulsions, the European and American Pharmacopeias assume only their visual inspection and droplet size evaluation, respectively [18,20].

All formulas had a pH close to physiological or slightly acidic (~6.00) compared to the pH of the blood. Among the tested PN admixtures, the relationship between pH and the content of phosphate ions was found. Despite the fact that the amino acid solution and other components contained in it show buffering properties, the influence of sodium glycerophosphate on the pH was observed in the studied PN admixtures. The administration of a PN admixture is often associated with metabolic complications, including metabolic acidosis. The main factors involved in the appearance of acid–base balance disorders during PN therapy are the metabolism of cationic amino acids and amino acids containing sulfuric acid, the titratable acidity of the PN admixtures, and the presence of acidificant agents (hydrochloric acid, acetic acid) in the PN admixture. Moreover, it was shown that hypophosphatemia that appears during PN therapy contributes significantly to the maintenance of metabolic acidosis [28]. In turn, increasing the dose of phosphates contributes to the reduction in PN admixture acidity (which was found in our study) and thus may further affect the acid–base balance in the patient’s body. 

After seven days of storage, the pH of all PN admixture variations did not change above 0.2 pH units. A significant drop in pH could indicate the decomposition of the lipid emulsion with the release of free fatty acids. In the PN admixtures selected for the study, no such phenomenon was observed. The osmolality of all PN admixtures was greater than 1000 mOsm/kg, meaning that these formulas can only be administered via the central veins [27,29]. The overall osmolality of a PN admixture depends on the osmolality of all its components and depends to the greatest extent on those components that have the highest content of osmotically active substances, in this case, electrolytes: sodium chloride and potassium chloride. Changes in the osmolality of the tested PN admixtures after seven days of storage did not exceed 4.4%, which proves that there were no changes in the concentration of osmotically active substances. 

The zeta potential is the electrokinetic potential that is observed at the surface of the dispersed droplets in contact with the electrolyte solution. The absolute value of zeta potential indicates the stability of the oil-in-water system. The zeta potential conversion from negative to positive charge indicates lipid emulsion breakdown. The dilution and addition of electrolytes during PN admixtures’ compounding affect the stability of lipid emulsion, increasing the values of zeta potential [27,30]. The results of the studied formulas after the storage period differed from those obtained upon preparation, but none of them underwent the charge conversion. 

The droplet size of the lipid emulsion, a critical parameter in the context of intravenous administration, was determined using the DLS method. According to chapter 729 of the American Pharmacopoeia, the DLS method is used to evaluate the droplet size distribution of lipid emulsions, and the MDD evaluated using this method cannot exceed 500 nm [20,31]. This method is appropriate only for samples characterized by PDI ranging from 0.05 to 0.7 [26]. The PDI of all variations of PN admixtures was in the range of 0.06 to 0.2, which proves the high homogeneity of the PN admixtures and that the DLS method is suitable for determining the droplet size. Lucchinetti et al. [32] state that the MDD of intravenous lipid emulsion depended on the brand names and ranged from 250 to 300 nm [32]. Our results showed that excluding Lipidem 20%, which was characterized by a lower MDD value, the studied lipid emulsions were in the range presented by Lucchinetti et al. [32]. The MDD of PN admixtures differed from the MDD obtained for pure lipid emulsions (Figure 2). Such results indicate that besides the type of lipid emulsion, the remaining ingredients of the PN admixtures also affect the lipid emulsion droplet size. Compounding of PN admixture and the conditions of its storage may affect the stability of the oil-in-water system, consequently leading to increased lipid droplet diameter. The administration of PN admixtures containing lipid droplets exceeding 500 nm may result in the obstruction of pulmonary arterioles or other small vessels by lipid droplet accumulation and/or liver inflammation by extending lipids uptake, since the size of lipid droplets determines their uptake [33]. Seki et al. [34] show, in the rat model, that about 30% of Intralipid with a droplet size of ~250 nm, compared to 80% of artificial lipoprotein-like particles with a droplet size ~50 nm, can be recovered after a single injection and liver passing. Thus, smaller lipid droplets may limit hepatic accumulation, potentially leading to less inflammation and metabolic derangements. Moreover, intracellular partitioning between fatty acid oxidation and lipid storage may also depend on the lipid droplet size [35]. 

The present study aimed to determine the long-term stability of Aminoplasmal Paed 10%-based pediatric PN admixtures. One of the limitations of this work is a lack of the addition of vitamin and trace elements into the PN admixtures. Vitamins show a particularly limited storage time due to their considerable susceptibility to degradation, which was shown in several studies, including those which performed HPLC analyses of selected vitamins during storage in different conditions [36,37,38]. The storage of PN admixtures containing vitamins and trace elements without chemical stability data is improper due to the degradation of the vitamins and the destabilizing impact of trace elements on the lipid emulsion. For this reason, it is a common clinical practice to add vitamins and trace elements immediately prior to the administration of the PN mixtures to the patients [39,40]. Another limitation concerns the determination of lipid emulsion droplet size based only on a dynamic light scattering method [20], supported by visual inspection. This is considered insufficient since the use of method II [20] and PFAT5 determination (the percentage of fat residing in globules larger than 5 µm) is superior as it allows for complete characterization of PN admixtures. However, such a methodology was previously used in several studies and deemed suitable and sufficient for droplet size determination [24,25,26,27]. 

It should also be mentioned that the methodology used in the presented study does not exclude the existence of incompatibilities in the PN admixture; however, it allows for the detection of those which are manifested by a change in the acid–base properties of the PN admixture, changes in the size of lipid emulsion droplets, and the occurrence of a precipitation process.

## 5. Conclusions

All studied PN admixtures based on Aminoplasmal Paed 10% were characterized by appropriate physicochemical parameters to be administered via the central veins, both immediately upon preparation and after seven days of storage at the temperature of 5 ± 1 °C with light protection. Electrolytes in used concentration ranges did not significantly affect the stability of PN admixtures, so they can be added to studied macronutrient composition. All variations of PN admixtures, regardless of the type of lipid emulsion used, were characterized by the MDD values within the pharmacopeial acceptance limit.

## Figures and Tables

**Table 1:** The concentration range of ingredients of PN admixtures.

Ingredients	Medicinal Product	Unit	Concentration Range
Amino acids	Aminoplasmal Paed 10%	g/L	17–21
Carbohydrates	Glucose 40%		123–150
Lipid emulsion *	Lipidem 20%/Clinoliec 20%/ Intralipid 20%/Smoflipid 20%		34–42
Sodium	Natrium chloratum 10%	mmol/L	3.53–97.93
Potassium	Kalium chloratum 15%		4.15–115.21
Magnesium	Inj. Magnesii Sulfurici 20%		1.98–10.28
Calcium	Calcio gluconato 1000 mg/10 mL		0.97–18.80
Phosphate	Glycophos 216 mg/mL		2.18–21.21

*—All PN variations were prepared using each of the following medicinal products: Lipidem 20% (B. Braun Melsungen AG, Melsungen, Germany), Clinoliec 20% (Baxter, Lessines, Belgium), Intralipid 20% (Fresenius Kabi AB, Upsala, Sweden), and Smoflipid 20% (Fresenius Kabi AB, Upsala, Sweden).

## Data Availability

Data are contained within the article.

## Supplementary Materials

The following are available online at https://www.mdpi.com/article/10.3390/pharmaceutics13071017/s1, Table S1: Composition of PN admixtures; Table S2: Physicochemical parameters of PN admixtures upon preparation (t = 0) and after seven days of storage (t = 7).
