# Supplementary material for: All-in-One Pediatric Parenteral Nutrition Admixtures with an Extended Shelf Life—Insight in Correlations between Composition and Physicochemical Parameters
Source: Pharmaceutics. 2021 Jul 2;13(7):1017. doi: 10.3390/pharmaceutics13071017 (PMC8309029; doi:10.3390/pharmaceutics13071017)
Supplement: Supplementary file 1 [file pharmaceutics-13-01017-s001.zip › pharmaceutics-1246583-supplementary.pdf]

pharmaceutics

MDPI

Supplementary Materials: All-In-One Pediatric Parenteral Nutrition Admixtures with an Extended Shelf Life—Insight in Correlations between Composition and Physicochemical Parameters

Aleksandra Gostyńska, Joanna Starkowska, Paulina Sobierajska, Anna Jelińska, Maciej Stawny

Table S1. Composition of PN admixtures

PN variations	Amino acids[1]	Glucose[2]	Lipid emulsion[3]	Sodium[4]	Potassium[5]	Magnesium[6]	Calcium[7]	Phosphate[8]
		[g/L]				[mmol/L]		
1	20.7	149.4	41.5	3.53	4.15	-	-	-
2	20.3	146.2	40.6	21.57	25.38	-	-	-
3	18.4	132.7	36.9	97.93	115.21	-	-	-
4	20.8	150.0	41.7	0	-	-	-	-
5	20.8	149.6	41.6	0	-	2.08	-	-
6	20.6	148.1	41.1	0	-	10.28	-	-
7	20.7	149.3	41.5	0	-	-	0.98	-
8	20.5	147.4	41.0	0	-	-	3.85	-
9	20.1	145.0	40.3	0	-	-	7.54	-
10	20.7	149.3	41.5	0	-	-	0.98	-
11	20.4	147.1	40.8	0	-	2.04	3.84	-
12	19.9	143.2	39.8	0	-	9.94	7.45	-
13	20.7	148.7	41.3	3.51	4.13	0.00	0.98	-
14	19.9	143.4	39.8	21.16	24.90	1.99	3.74	-
15	17.7	127.3	35.4	93.96	110.54	8.84	6.63	-
16	20.8	149.7	41.6	4.36	-	-	-	2.18
17	20.7	149.3	41.5	8.72	-	-	-	4.36
18	20.4	146.8	40.8	42.42	-	-	-	21.21
19	20.6	148.4	41.2	7.82	4.12	0.00	0.97	2.16
20	19.8	142.8	39.7	29.41	24.79	1.98	3.73	4.17
21	17.4	125.0	34.7	128.38	108.54	8.68	6.51	18.06
22	20.5	147.5	41.0	14.11	16.60	-	-	-
23	19.8	142.6	39.6	43.15	50.76	-	-	-
24	18.9	135.8	37.7	78.34	92.17	-	-	-
25	20.8	149.6	41.6	0	-	2.08	-	-
26	20.7	149.2	41.5	0	-	4.16	-	-
27	20.6	148.5	41.2	0	-	8.23	-	-
28	20.7	148.8	41.3	0	-	-	1.87	-
29	20.0	143.7	39.9	0	-	-	9.68	-
30	19.2	137.9	38.3	0	-	-	19.03	-
31	20.6	148.4	41.2	0	-	2.07	1.87	-
32	19.9	143.0	39.7	0	-	4.08	9.65	-
33	19.0	136.6	38.0	0	-	7.95	18.79	-
34	20.3	146.0	40.5	14.05	16.53	2.07	1.86	-
35	18.9	136.2	37.8	42.32	49.79	3.98	9.41	-
36	17.3	124.8	34.7	75.17	88.43	7.07	16.71	-
37	20.8	149.6	41.6	5.2	-	-	-	2.60
38	20.7	148.8	41.3	16.6	-	-	-	8.30
39	20.5	147.5	41.0	32.62	-	-	-	16.31
40	20.2	145.6	40.4	19.18	16.49	2.06	1.86	2.58
41	18.8	135.2	37.6	58.01	49.58	3.97	9.37	7.93
42	17.1	123.1	34.2	101.59	86.84	6.95	16.41	13.89

[1] – the source of amino acids was Aminoplasmal Paed 10% (B. Braun Melsungen AG, Melsungen, Germany)

[2] – the source of glucose was Glucose 40% (B. Braun Melsungen AG, Melsungen, Germany)

[3] - all PN variations were prepared using each of the following medicinal products: Lipidem 20% (B. Braun Melsungen AG, Melsungen, Germany), Clinoliec 20% (Baxter, Lessines, Belgium), Intralipid 20% (Fresenius Kabi AB. Upsala. Sweden), and Smoflipid 20% (Fresenius Kabi AB, Upsala, Sweden)

[4] – the source of sodium was Natrium chloratum 10% ((B. Braun Melsungen AG, Melsungen, Germany)

[5] – the source of potassium was Kalium chloratum 15% (WZF Polfa S.A., Warsaw, Poland)

[6] – the source of magnesium was Inj. Magnesii Sulfurici 20% (WZF Polfa S.A., Warsaw, Poland)

[7] – the source of calcium was Calcio gluconato 1000 mg/10mL (Added Pharma, Netherlands)

[8] – the source of phosphate was Glycophos 216 mg/mL (Fresenius Kabi AB, Upsala, Sweden)

Table S2. Physicochemical parameters of PN admixtures upon preparation (t = 0) and after seven days of storage (t = 7).

Formulation		MDD [nm]		PDI		DV(90) [nm]		pH		Osmolality [mOsm/kg]		Potential zeta [mV]	
		t = 0	t = 7	t = 0	t = 7	t = 0	t = 7	t = 0	t = 7	t = 0	t = 7	t = 0	t = 7
PN 1	1	241.40 ± 5.63	245.50 ± 2.10	0.07 ± 0.02	0.10 ± 0.02	346.67 ± 14.01	362.67 ± 11.02	5.99 ± 0.01	5.98 ± 0.01	1366 ± 14	1366 ± 15	−36.53 ± 0.40	−33.07 ± 2.20
PN 1	2	247.30 ± 1.74	244.60 ± 0.26	0.08 ± 0.01	0.07 ± 0.01	367.67 ± 5.03	352.33 ± 10.97	5.96 ± 0.00	5.96 ± 0.01	1428 ± 4	1437 ± 3	31.53 ± 1.00	−19.40 ± 0.62
PN 1	3	246.90 ± 6.08	248.07 ± 6.86	0.12 ± 0.03	0.09 ± 0.02	374.00 ± 16.52	365.00 ± 22.11	5.94 ± 0.00	5.94 ± 0.01	1650 ± 1	1670 ± 2	−18.07 ± 0.32	−18.40 ± 0.44
PN 1	4	245.10 ± 3.10	247.50 ± 4.86	0.07 ± 0.03	0.06 ± 0.01	355.67 ± 15.28	359.33 ± 19.66	6.07 ± 0.00	6.03 ± 0.02	1373 ± 4	1375 ± 3	−38.63 ± 0.25	−29.27 ± 0.76
PN 1	5	247.13 ± 9.96	247.67 ± 1.30	0.09 ± 0.02	0.09 ± 0.01	365.33 ± 29.84	371.33 ± 5.86	6.00 ± 0.00	5.97 ± 0.00	1378 ± 0	1366 ± 2	−23.50 ± 0.26	−23.00 ± 0.72
PN 1	6	249.03 ± 2.76	241.17 ± 3.28	0.08 ± 0.02	0.07 ± 0.01	363.67 ± 16.50	340.33 ± 5.69	5.96 ± 0.00	5.95 ± 0.00	1369 ± 1	1360 ± 3	−14.53 ± 0.32	−13.20 ± 0.50
PN 1	7	251.67 ± 6.94	245.63 ± 8.22	0.09 ± 0.02	0.09 ± 0.01	371.67 ± 21.36	359.67 ± 23.86	5.95 ± 0.00	5.97 ± 0.01	1374 ± 3	1377 ± 3	−28.10 ± 0.36	−29.87 ± 0.59
PN 1	8	241.70 ± 1.31	245.33 ± 4.73	0.09 ± 0.03	0.08 ± 0.02	345.67 ± 11.55	352.67 ± 16.07	5.93 ± 0.00	5.96 ± 0.01	1361 ± 2	1361 ± 1	−19.97 ± 0.15	−18.93 ± 0.55
PN 1	9	249.43 ± 6.09	247.87 ± 12.59	0.09 ± 0.01	0.12 ± 0.02	364.67 ± 11.15	371.67 ± 30.02	5.94 ± 0.00	5.96 ± 0.00	1344 ± 8	1341 ± 5	−15.30 ± 0.61	−14.57 ± 0.81
PN 1	10	253.27 ± 3.41	242.37 ± 3.84	0.09 ± 0.01	0.06 ± 0.01	378.67 ± 3.51	343.00 ± 7.00	5.95 ± 0.01	5.95 ± 0.01	1370 ± 4	1376 ± 3	−27.47 ± 0.61	−25.00 ± 0.46
PN 1	11	250.53 ± 12.34	255.30 ± 4.20	0.10 ± 0.04	0.10 ± 0.01	373.67 ± 30.73	388.00 ± 9.64	5.93 ± 0.00	5.94 ± 0.01	1336 ± 10	1360 ± 1	−17.23 ± 0.06	−16.73 ± 0.45
PN 1	12	247.73 ± 10.28	246.40 ± 2.55	0.09 ± 0.04	0.09 ± 0.02	356.00 ± 28.58	363.33 ± 7.09	5.94 ± 0.01	5.94 ± 0.01	1329 ± 3	1337 ± 8	−9.28 ± 0.27	−10.18 ± 0.33
PN 1	13	252.07 ± 7.33	253.47 ± 3.40	0.10 ± 0.01	0.10 ± 0.00	376.67 ± 15.14	384.33 ± 8.14	5.94 ± 0.00	5.95 ± 0.00	1397 ± 14	1386 ± 2	−25.50 ± 0.52	−25.97 ± 0.76
PN 1	14	252.60 ± 1.56	242.37 ± 6.01	0.09 ± 0.02	0.08 ± 0.03	380.33 ± 10.21	349.33 ± 19.86	5.91 ± 0.00	5.91 ± 0.00	1419 ± 7	1422 ± 1	−15.80 ± 0.20	−15.53 ± 0.38
PN 1	15	242.30 ± 2.07	246.73 ± 1.89	0.06 ± 0.02	0.10 ± 0.01	347.00 ± 10.44	365.33 ± 7.77	5.92 ± 0.01	5.91 ± 0.00	1614 ± 5	1618 ± 7	−8.63 ± 0.55	−28.30 ± 0.69
PN 1	16	242.17 ± 5.99	243.47 ± 5.98	0.08 ± 0.01	0.07 ± 000	351.33 ± 17.16	348.33 ± 16.01	6.23 ± 0.00	6.23 ± 0.00	1382 ± 3	1381 ± 7	−34.33 ± 0.32	−40.57 ± 0.76
PN 1	17	252.50 ± 3.14	244.33 ± 5.10	0.10 ± 0.01	0.08 ± 0.02	383.33 ± 2.89	352.33 ± 20.65	6.39 ± 0.00	6.41 ± 0.01	1376 ± 3	1390 ± 0	−36.83 ± 0.95	−39.73 ± 0.31
PN 1	18	243.73 ± 5.95	245.90 ± 7.71	0.07 ± 0.01	0.08 ± 0.03	352.00 ± 15.39	358.00 ± 24.43	6.89 ± 0.00	6.93 ± 0.00	1402 ± 2	1402 ± 10	−41.30 ± 1.71	−40.93 ± 0.65

PN 1	19	243.13 ± 5.13	246.87 ± 6.41	0.08 ± 0.04	0.08 ± 0.01	348.67 ± 16.20	363.00 ± 21.17	6.21 ± 0.01	6.22 ± 0.02	1399 ± 10	1382 ± 7	−24.60 ± 0.10	−27.57 ± 0.67
PN 1	20	246.57 ± 4.80	246.37 ± 3.79	0.09 ± 0.01	0.10 ± 0.01	365.33 ± 11.93	364.00 ± 12.53	6.29 ± 0.01	6.29 ± 0.00	1428 ± 4	1419 ± 4	−16.53 ± 0.47	−17.40 ± 0.79
PN 1	21	240.73 ± 4.04	241.80 ± 2.85	0.09 ± 0.02	0.08 ± 0.02	350.67 ± 16.07	353.67 ± 11.02	6.65 ± 0.01	6.65 ± 0.00	1626 ± 3	1642 ± 3	−13.17 ± 0.21	−13.37 ± 0.64
PN 1	22	244.73 ± 1.30	251.63 ± 5.00	0.07 ± 0.02	0.11 ± 0.01	352.00 ± 6.56	371.00 ± 16.70	5.98 ± 0.02	5.99 ± 0.00	1458 ± 5	1453 ± 19	−29.73 ± 0.35	−18.33 ± 2.64
PN 1	23	247.53 ± 6.12	248.93 ± 1.30	0.10 ± 0.01	0.09 ± 0.01	365.33 ± 21.13	367.33 ± 4.93	5.97 ± 0.01	5.99 ± 0.01	1501 ± 6	1501 ± 3	−26.17 ± 0.51	−21.00 ± 0.70
PN 1	24	249.93 ± 3.09	248.33 ± 2.41	0.10 ± 0.01	0.1 ± 0.01	375.00 ± 11.79	365.33 ± 1.53	5.98 ± 0.01	5.98 ± 0.00	1609 ± 5	1597 ± 5	−21.73 ± 2.51	−17.53 ± 2.36
PN 1	25	245.60 ± 6.94	244.00 ± 3.81	0.07 ± 0.05	0.08 ± 0.02	360.67 ± 29.26	355.33 ± 13.58	6.03 ± 0.01	6.04 ± 0.00	1373 ± 7	1369 ± 10	−30.93 ± 0.64	−14.30 ± 0.30
PN 1	26	241.13 ± 2.48	250.83 ± 2.60	0.08 ± 0.01	0.1 ± 0.02	348.67 ± 12.66	374.33 ± 14.29	6.01 ± 001	6.03 ± 0.01	1371 ± 0	1365 ± 11	−19.93 ± 0.72	−18.73 ± 0.45
PN 1	27	247.37 ± 2.25	254.33 ± 6.05	0.08 ± 0.00	0.08 ± 0.01	367.33 ± 9.61	377.00 ± 12.77	5.99 ± 0.00	6.01 ± 0.01	1368 ± 6	1363 ± 1	−20.87 ± 0.31	-14.40 ± 0.46
PN 1	28	245.87 ± 2.70	251.67 ± 3.09	0.09 ± 0.02	0.09 ± 0.01	361.33 ± 11.55	377.00 ± 8.54	6.02 ± 0.00	6.05 ± 0.00	1361 ± 2	1366 ± 5	−24.27 ± 0.47	−23.37 ± 0.12
PN 1	29	242.53 ± 4.02	249.47 ± 5.93	0.08 ± 0.03	0.09 ± 0.01	354.00 ± 20.22	365.67 ± 14.64	6.02 ± 000	6.05 ± 0.01	1333 ± 7	1332 ± 8	−11.47 ± 0.31	−10.43 ± 0.21
PN 1	30	246.33 ± 6.57	242.37 ± 2.55	0.08 ± 0.03	0.07 ± 0.01	360.33 ± 24.85	350.33 ± 15.95	6.02 ± 0.01	6.05 ± 0.00	1285 ± 3	1291 ± 15	−6.59 ± 0.20	−6.97 ± 0.14
PN 1	31	253.60 ± 3.35	247.30 ± 6.46	0.11 ± 0.01	0.08 ± 0.02	388.00 ± 9.00	361.67 ± 28.59	6.00 ± 0.00	6.03 ± 0.00	1355 ± 7	1357 ± 2	−19.17 ± 0.46	−13.90 ± 0.26
PN 1	32	251.57 ± 6.13	247.30 ± 7.73	0.10 ± 0.01	0.08 ± 0.02	376.00 ± 23.39	363.33 ± 20.26	6.00 ± 0.00	6.04 ± 0.00	1323 ± 3	1329 ± 3	−9.19 ± 0.10	−9.75 ± 0.49
PN 1	33	245.10 ± 2.65	247.27 ± 5.42	0.09 ± 0.01	0.08 ± 0.02	362.33 ± 10.02	360.00 ± 19.16	6.01 ± 0.00	6.04 ± 0.01	1272 ± 3	1269 ± 1	−5.94 ± 0.44	−5.10 ± 0.49
PN 1	34	246.07 ± 1.46	252.90 ± 9.11	0.10 ± 0.01	0.1 ± 0.02	362.00 ± 5.29	384.00 ± 31.00	5.98 ± 0.01	5.99 ± 0.00	1398 ± 3	1397 ± 1	−18.07 ± 0.45	−15.30 ± 0.79
PN 1	35	239.80 ± 4.28	247.33 ± 3.66	0.08 ± 0.03	0.1 ± 0.01	346.00 ± 13.11	369.33 ± 9.45	5.98 ± 0.01	5.99 ± 0.00	1453 ± 10	1443 ± 3	−8.99 ± 0.19	−8.94 ± 0.76
PN 1	36	243.90 ± 4.20	244.37 ± 6.93	0.07 ± 0.01	0.08 ± 0.02	355.33 ± 15.50	357.00 ± 24.58	5.99 ± 0.01	6.00 ± 0.00	1509 ± 2	1504 ± 1	−5.68 ± 0.32	−5.01 ± 0.52
PN 1	37	249.97 ± 0.35	242.03 ± 3.20	0.10 ± 0.01	0.06 ± 0.01	377.00 ± 7.55	343.00 ± 13.45	6.33 ± 0.00	6.34 ± 0.02	1380 ± 1	1388 ± 8	−37.17 ± 0.71	−14.47 ± 0.49
PN 1	38	247.63 ± 4.80	248.10 ± 2.54	0.10 ± 0.03	0.07 ± 0.02	364.33 ± 21.13	365.00 ± 2.65	6.68 ± 0.01	6.70 ± 0.01	1377 ± 3	1391 ± 2	−38.00 ± 0.96	−16.73 ± 0.25
PN 1	39	246.73 ± 2.72	246.17 ± 2.22	0.08 ± 0.03	0.08 ± 0.01	365.67 ± 15.14	358.67 ± 17.21	6.92 ± 0.01	6.96 ± 0.00	1385 ± 6	1408 ± 0	−40.30 ± 1.04	−38.37 ± 0.49
PN 1	40	246.93 ± 3.19	246.97 ± 7.14	0.08 ± 0.01	0.09 ± 0.02	362.33 ± 12.42	361.33 ± 19.73	6.26 ± 0.00	6.29 ± 0.00	1435 ± 4	1415 ± 8	−21.00 ± 0.35	−20.53 ± 0.60
PN 1	41	242.87 ± 4.41	242.27 ± 1.04	0.09 ± 0.01	0.06 ± 0.01	375.00 ± 2.00	344,00 ± 2.65	6.48 ± 0.01	6.50 ± 0.00	1481 ± 5	1471 ± 7	−13.13 ± 0.74	−12.83 ± 0.47
PN 1	42	250.33 ± 2.12	242.47 ± 4.75	0.08 ± 0.02	0.06 ± 0.02	354.67 ± 18.50	346.67 ± 5.13	6.65 ± 0.00	6.64 ± 0.01	1543 ± 18	1539 ± 4	−9.15 ± 0.13	−9.10 ± 0.22
PN 2	1	279.23 ± 4.22	293.53 ± 2.46	0.07 ± 0.02	0.11 ± 0.02	403.67 ± 15.95	446.67 ± 9.29	6.00 ± 0.01	6.02 ± 0.01	1362 ± 1	1334 ± 3	−16.13 ± 0.21	−16.60 ± 0.53
PN 2	2	295.53 ± 9.15	292.93 ± 5.61	0.11 ± 0.01	0.10 ± 0.01	450.00 ± 26.06	439.00 ± 10.44	5.95 ± 0.02	5.99 ± 0.00	1416 ± 1	1398 ± 2	−23.30 ± 2.39	−20.97 ± 0.15
PN 2	3	285.9 ± 3.99	292.70 ± 4.92	0.07 ± 0.02	0.08 ± 0.01	416.00 ± 13.00	428.00 ± 11.79	5.95 ± 0.00	5.99 ± 0.01	1635 ± 2	1614 ± 3	−12.17 ± 0.55	−12.63 ± 0.51
PN 2	4	294.03 ± 3.76	291.23 ± 4.68	0.10 ± 0.01	0.10 ± 0.02	443.33 ± 10.79	436.00 ± 15.72	6.04 ± 0.00	6.08 ± 0.01	1359 ± 6	1348 ± 8	−26.80 ± 0.30	−26.70 ± 0.10
PN 2	5	291.87 ± 1.36	295.93 ± 8.47	0.11 ± 0.01	0.11 ± 0.01	439.33 ± 4.04	446.00 ± 14.00	5.98 ± 0.00	6.02 ± 0.01	1352 ± 2	1342 ± 9	−17.90 ± 0.53	−11.63 ± 0.38
PN 2	6	298.43 ± 2.95	300.47 ± 7.02	0.12 ± 0.01	0.11 ± 0.02	457.00 ± 7.00	461.67 ± 23.07	5.96 ± 0.00	5.99 ± 0.00	1360 ± 2	1351 ± 6	−13.30 ± 0.46	−9.97 ± 0.24
PN 2	7	294.00 ± 9.73	291.63 ± 5.80	0.10 ± 0.03	0.09 ± 0.02	446.67 ± 28.68	430.67 ± 20.26	5.98 ± 0.01	6.02 ± 0.00	1353 ± 1	1340 ± 3	−21.93 ± 0.55	−18.37 ± 0.84

PN 2	8	289.33 ± 8.43	297.87 ± 4.30	0.09 ± 0.03	0.10 ± 0.01	432.00 ± 23.81	450.33 ± 12.58	5.97 ± 0.01	6.01 ± 0.00	1343 ± 2	1325 ± 2	−13.13 ± 0.55	−13.43 ± 0.40
PN 2	9	292.03 ± 6.29	295.53 ± 5.00	0.12 ± 001	0.10 ± 0.01	447.67 ± 11.59	444.33 ± 20.50	5.98 ± 0.00	6.01 ± 0.00	1326 ± 1	1308 ± 1	−12.07 ± 0.55	−9.62 ± 0.31
PN 2	10	291.60 ± 5.07	294.13 ± 4.11	0.10 ± 0.03	0.10 ± 0.01	438.67 ± 16.17	439.67 ± 12.42	5.97 ± 0.00	6.01 ± 0.00	1359 ± 3	1346 ± 3	−25.17 ± 0.86	−19.33 ± 0.40
PN 2	11	298.63 ± 4.64	296.60 ± 11.23	0.11 ± 0.03	0.11 ± 0.01	459.00 ± 25.12	447.33 ± 28.73	5.97 ± 0.00	5.99 ± 0.01	1345 ± 1	1334 ± 7	−11.20 ± 0.17	−11.80 ± 0.75
PN 2	12	291.47 ± 8.78	293.23 ± 3.33	0.10 ± 0.02	0.07 ± 0.02	438.33 ± 25.54	428.67 ± 17.47	5.97 ± 0.00	5.99 ± 0.01	1331 ± 4	1311 ± 0	−5.23 ± 0.41	−5.70 ± 0.38
PN 2	13	293.77 ± 3.62	296.03 ± 5.24	0.11 ± 0.01	0.09 ± 0.02	445.33 ± 12.10	440.33 ± 12.50	5.96 ± 0.01	5.99 ± 0.00	1373 ± 1	1342 ± 5	−19.77 ± 0.70	−18.40 ± 0.79
PN 2	14	287.83 ± 1.33	294.87 ± 5.75	0.07 ± 0.02	0.10 ± 0.02	413.00 ± 15.59	441.00 ± 17.58	5.94 ± 0.00	5.96 ± 0.01	1416 ± 1	1381 ± 2	−9.88 ± 0.27	−10.50 ± 0.10
PN 2	15	299.50 ± 4.79	294.47 ± 4.21	0.14 ± 0.02	0.10 ± 0.01	467.00 ± 17.58	443.33 ± 0.58	5.94 ± 0.01	5.97 ± 0.01	1607 ± 1	1591 ± 2	−4.61 ± 0.25	−5.21 ± 1.00
PN 2	16	294.47 ± 4.65	300.07 ± 7.95	0.11 ± 0.01	0.11 ± 0.02	449.33 ± 15.31	459.33 ± 23.03	6.23 ± 0.00	6.29 ± 0.02	1371 ± 6	1347 ± 2	−11.87 ± 0.32	−24.10 ± 0.66
PN 2	17	300.97 ± 9.60	297.43 ± 4.22	0.12 ± 0.03	0.12 ± 0.02	471.00 ± 34.39	454.33 ± 13.32	6.38 ± 0.00	6.47 ± 0.00	1368 ± 3	1353 ± 0	−11.20 ± 0.46	−21.63 ± 0.81
PN 2	18	293.53 ± 2.66	290.37 ± 8.31	0.10 ± 0.01	0.09 ± 0.01	440.00 ± 3.00	427.33 ± 24.66	6.94 ± 0.01	7.01 ± 0.01	1388 ± 8	1373 ± 1	−28.50 ± 0.44	−30.63 ± 0.76
PN 2	19	301.40 ± 3.86	297.37 ± 9.86	0.13 ± 0.01	0.11 ± 0.01	476.67 ± 17.62	455.00 ± 25.63	6.21 ± 0.01	6.31 ± 0.00	1363 ± 10	1359 ± 6	−23.03 ± 0.15	−21.93 ± 0.06
PN 2	20	291.77 ± 1.44	287.43 ± 8.89	0.10 ± 0.00	0.08 ± 0.04	440.33 ± 5.86	420.67 ± 30.66	6.34 ± 0.01	6.34 ± 0.00	1409 ± 0	1395 ± 1	−13.57 ± 0.15	−13.07 ± 0.40
PN 2	21	284.93 ± 5.78	287.47 ± 7.92	0.09 ± 0.02	0.08 ± 0.02	422.00 ± 16.70	420.00 ± 23.39	6.60 ± 0.00	6.70 ± 0.01	1622 ± 5	1613 ± 7	−8.78 ± 0.22	−9.53 ± 0.37
PN 2	22	287.77 ± 2.55	290.77 ± 1.76	0.10 ± 0.05	0.09 ± 0.01	425.33 ± 21.22	433.00 ± 7.81	6.00 ± 0.00	6.02 ± 0.01	1400 ± 7	1389 ± 11	−15.93 ± 0.65	−21.67 ± 0.50
PN 2	23	283.10 ± 3.31	288.67 ± 7.77	0.10 ± 0.03	0.10 ± 0.03	426.67 ± 12.06	429.33 ± 28.94	5.99 ± 0.01	5.99 ± 0.00	1478 ± 1	1483 ± 1	−11.03 ± 0.42	−16.33 ± 1.07
PN 2	24	294.07 ± 3.48	288.07 ± 7.65	0.10 ± 0.01	0.11 ± 0.02	446.33 ± 6.35	434.00 ± 28.16	5.98 ± 0.01	5.99 ± 0.01	1594 ± 12	1606 ± 1	−15.57 ± 2.22	−14.43 ± 1.50
PN 2	25	297.67 ± 5.71	287.07 ± 4.03	0.11 ± 0.01	0.10 ± 0.01	459.33 ± 18.18	429.33 ± 6.43	6.06 ± 0.01	6.04 ± 0.01	1367 ± 1	1362 ± 1	−12.37 ± 0.06	−20.17 ± 0.55
PN 2	26	283.33 ± 1.80	291.53 ± 5.70	0.09 ± 0.02	0.11 ± 0.02	417.33 ± 19.14	443.00 ± 19.92	6.06 ± 0.01	6.03 ± 0.00	1363 ± 1	1362 ± 5	−16.10 ± 0.36	−14.70 ± 0.46
PN 2	27	295.90 ± 8.49	286.40 ± 0.87	0.10 ± 0.01	0.11 ± 0.03	449.67 ± 24.42	432.67 ± 6.66	6.02 ± 0.01	6.01 ± 0.01	1363 ± 1	1374 ± 4	−16.33 ± 0.38	−10.25 ± 0.49
PN 2	28	291.47 ± 8.86	289.20 ± 5.04	0.10 ± 0.02	0.09 ± 0.03	434.67 ± 26.86	431.00 ± 15.52	6.02 ± 0.01	6.05 ± 0.00	1357 ± 4	1366 ± 3	−16.70 ± 0.10	−18.60 ± 0.35
PN 2	29	290.90 ± 9.54	286.10 ± 2.21	0.09 ± 0.03	0.08 ± 0.02	430.67 ± 31.21	420.33 ± 15.28	6.02 ± 0.00	6.04 ± 0.00	1311 ± 2	1328 ± 5	−7.73 ± 0.29	−9.55 ± 0.40
PN 2	30	292.73 ± 8.30	292.70 ± 3.12	0.10 ± 0.01	0.11 ± 0.01	442.33 ± 23.71	445.67 ± 4.73	6.04 ± 0.00	6.05 ± 0.00	1258 ± 5	1291 ± 4	−4.36 ± 0.48	−4.51 ± 0.17
PN 2	31	287.47 ± 3.43	287.70 ± 7.13	0.11 ± 0.01	0.09 ± 0.02	431.00 ± 2.,38	430.67 ± 22.50	6.02 ± 0.00	6.03 ± 0.00	1349 ± 1	1373 ± 6	−15.00 ± 0.62	−17.27 ± 0.21
PN 2	32	286.47 ± 3.46	295.83 ± 5.63	0.09 ± 0.00	0.12 ± 0.01	425.33 ± 6.43	458.33 ± 14.64	6.02 ± 0.00	6.03 ± 0.00	1311 ± 1	1320 ± 3	−5.92 ± 0.14	−7.31 ± 0.30
PN 2	33	292.27 ± 4.13	296.67 ± 6.16	0.11 ± 0.02	0.11 ± 0.02	437.33 ± 12.42	455.67 ± 23.86	6.05 ± 0.00	6.05 ± 0.00	1238 ± 2	1292 ± 6	−2.33 ± 0.35	−3.76 ± 0.16
PN 2	34	291.93 ± 1.63	286.90 ± 8.43	0.12 ± 0.02	0.10 ± 0.02	448.00 ± 3.46	426.67 ± 32.72	5.99 ± 0.01	6.00 ± 0.00	1399 ± 1	1401 ± 12	−13.53 ± 0.12	−12.33 ± 0.40
PN 2	35	289.77 ± 7.75	288.03 ± 4.04	0.08 ± 0.01	0.10 ± 0.02	427.33 ± 21.50	432.00 ± 19.08	5.99 ± 0.01	5.99 ± 0.00	1428 ± 6	1448 ± 3	−6.16 ± 0.43	−5.89 ± 0.15
PN 2	36	283.90 ± 2.01	288.50 ± 3.41	0.09 ± 0.00	0.10 ± 0.01	415.00 ± 2.00	434.00 ± 15.59	6.00 ± 0.01	6.00 ± 0.02	1508 ± 3	1475 ± 2	−2.16 ± 0.41	−2.32 ± 0.91
PN 2	37	294.53 ± 5.84	289.60 ± 6.80	0.12 ± 0.02	0.10 ± 0.01	448.67 ± 14.47	433.67 ± 25.74	6.34 ± 0.00	6.35 ± 0.00	1374 ± 1	1369 ± 2	−23.00 ± 0.17	−25.30 ± 0.10
PN 2	38	287.47 ± 1.60	288.50 ± 5.97	0.10 ± 0.01	0.09 ± 0.04	424.67 ± 2.08	430.67 ± 21.78	6.67 ± 0.00	6.68 ± 0.01	1386 ± 15	1378 ± 3	−26.33 ± 0.91	−32.13 ± 0.91

PN 2	39	295.03 ± 4.72	287.13 ± 6.21	0.10 ± 0.01	0.12 ± 0.01	444.33 ± 11.50	439.33 ± 13.05	6.92 ± 0.00	6.94 ± 0.00	1392 ± 1	1409 ± 24	−31.17 ± 1.11	−30.63 ± 1.25
PN 2	40	284.77 ± 3.18	292.57 ± 2.40	0.11 ± 0.02	0.09 ± 0.03	420.33 ± 18.58	439.00 ± 9.54	6.28 ± 0.01	6.31 ± 0.00	1388 ± 8	1390 ± 11	−1407 ± 0.38	−15.43 ± 0.74
PN 2	41	287.57 ± 1.11	297.13 ± 6.37	0.09 ± 0.01	0.12 ± 0.03	424.67 ± 7.23	465.33 ± 25.70	6.47 ± 0.01	6.51 ± 0.01	1461 ± 16	1441 ± 2	−9.25 ± 0.44	−8.60 ± 0.68
PN 2	42	289.73 ± 4.21	288.13 ± 4.91	0.09 ± 0.01	0.10 ± 0.01	430.00 ± 13.45	433.33 ± 14.64	6.62 ± 0.01	6.66 ± 0.01	1518 ± 1	1501 ± 3	−5.91 ± 0.39	−6.17 ± 0.56
PN 3	1	344.13 ± 8.26	329.17 ± 5.10	0.13 ± 0.01	0.12 ± 0.03	542.67 ± 27.43	498.67 ± 11.93	5.99 ± 0.00	6.00 ± 0.01	1392 ± 0	1383 ± 15	−28.20 ± 0.89	−27.40 ± 0.96
PN 3	2	337.93 ± 1.05	348.87 ± 9.36	0.10 ± 0.03	0.14 ± 0.03	510.67 ± 17.62	548.33 ± 37.82	5.95 ± 0.00	5.96 ± 0.01	1452 ± 3	1448 ± 4	−23.03 ± 0.84	−20.40 ± 1.21
PN 3	3	335.53 ± 15.83	338.83 ± 5.46	0.08 ± 0.04	0.11 ± 0.01	504.33 ± 52.97	512.00 ± 9.17	5.93 ± 0.01	5.96 ± 0.00	1658 ± 7	1657 ± 1	−13.87 ± 0.55	−15.73 ± 0.67
PN 3	4	347.00 ± 7.79	342.87 ± 6.45	0.12 ± 0.02	0.11 ± 0.01	546.67 ± 32.58	518.67 ± 21.50	6.03 ± 0.01	6.06 ± 0.00	1387 ± 8	1387 ± 5	−29.17 ± 0.31	−20.50 ± 0.78
PN 3	5	332.33 ± 5.16	341.60 ± 6.36	0.11 ± 0.02	0.11 ± 0.02	501.67 ± 14.43	525.67 ± 25.54	5.98 ± 0.00	6.00 ± 0.01	1385 ± 1	1386 ± 1	−25.40 ± 1.21	−16.43 ± 0.25
PN 3	6	348.33 ± 3.84	349.87 ± 11.05	0.11 ± 0.02	0.14 ± 0.03	541.33 ± 25.77	555.67 ± 33.01	5.95 ± 0.00	5.97 ± 0.01	1387 ± 3	1390 ± 2	−10.50 ± 0.36	−11.50 ± 0.10
PN 3	7	346.70 ± 10.69	347.53 ± 15.07	0.13 ± 0.01	0.12 ± 0.04	547.33 ± 33.49	542.33 ± 58.60	5.97 ± 0.01	6.01 ± 0.00	1389 ± 4	1401 ± 2	−23.93 ± 0.58	−23.87 ± 0.64
PN 3	8	334.17 ± 16.28	343.67 ± 3.45	0.10 ± 0.03	0.12 ± 0.01	508.67 ± 51.54	523.33 ± 8.02	5.97 ± 0.00	6.00 ± 0.00	1375 ± 5	1373 ± 1	−14.67 ± 0.40	−16.27 ± 0.40
PN 3	9	352.47 ± 13.86	342.50 ± 6.33	0.12 ± 0.02	0.11 ± 0.03	552.00 ± 40.63	528.00 ± 2425	5.98 ± 0.00	6.00 ± 0.00	1352 ± 1	1351 ± 2	−10.53 ± 0.32	−11.37 ± 0.57
PN 3	10	349.70 ± 3.39	335.03 ± 2.75	0.13 ± 0.01	0.12 ± 0.04	557.67 ± 4.73	513.00 ± 18.68	5.97 ± 0.01	6.02 ± 0.00	1389 ± 4	1389 ± 11	−22.37 ± 0.55	−21.97 ± 0.45
PN 3	11	335.70 ± 15.10	345.03 ± 3,.6	0.11 ± 0.03	0.12 ± 0.02	501.00 ± 49.24	528.67 ± 10.60	5.96 ± 0.00	5.99 ± 0.00	1360 ± 10	1366 ± 12	−12.13 ± 0.35	−13.97 ± 0.25
PN 3	12	331.63 ± 10.42	342.80 ± 6.68	0.12 ± 0.01	0.11 ± 0.02	503.33 ± 25.03	523.00 ± 22.52	5.98 ± 0.00	6.00 ± 0.00	1348 ± 11	1346 ± 2	−5.53 ± 0.33	−6.80 ± 0.12
PN 3	13	342.30 ± 8.52	342.97 ± 11.35	0.12 ± 0.02	0.14 ± 0.03	528.67 ± 21.78	534.00 ± 21.28	5.96 ± 0.00	5.99 ± 0.01	1377 ± 23	1405 ± 6	−21.23 ± 0.46	−22.03 ± 0.21
PN 3	14	339.27 ± 15.52	343.67 ± 3.84	0.13 ± 0.03	0.10 ± 0.01	530.33 ± 27.43	523.33 ± 14.74	5.91 ± 0.01	5.96 ± 0.00	1431 ± 3	1440 ± 1	−11.17 ± 0.25	−12.70 ± 0.40
PN 3	15	332.53 ± 4.65	348.10 ± 14.17	0.08 ± 0.01	0.12 ± 0.01	494.33 ± 8.50	540.33 ± 41.79	5.91 ± 0.01	5.98 ± 0.01	1678 ± 7	1651 ± 0	−6.03 ± 0.13	−6.29 ± 0.25
PN 3	16	341.47 ± 16.75	339.47 ± 13.20	0.12 ± 0.03	0.13 ± 0.01	532.00 ± 57.30	520.00 ± 32.14	6.26 ± 0.00	6.32 ± 0.00	1396 ± 2	1390 ± 6	−27.93 ± 0.25	−25.40 ± 0.82
PN 3	17	335.13 ± 2.05	339.17 ± 8.50	0.11 ± 0.01	0.10 ± 0=03	509.33 ± 8.02	514.00 ± 35.04	6.42 ± 0.00	6.50 ± 0.01	1396 ± 3	1394 ± 0	−16.17 ± 0.21	−21.67 ± 0.49
PN 3	18	335.03 ± 3.04	351.00 ± 13.91	0.10 ± 0.01	0..14 ± 0.01	507.00 ± 11.00	562.00 ± 34.04	7.00 ± 0.00	7.04 ± 0.01	1424 ± 1	1421 ± 4	−29.30 ± 0.61	−32.87 ± 0.38
PN 3	19	339.50 ± 14.78	328.43 ± 12.11	0.13 ± 0.01	0.15 ± 0.02	525.00 ± 39.69	503.00 ± 17.35	6.27 ± 0.04	6.29 ± 0.00	1399 ± 4	1400 ± 9	−24.10 ± 0.66	−26.00 ± 0.36
PN 3	20	337.30 ± 2.55	341.97 ± 9.60	0.11 ± 0.02	0.10 ± 0.03	512.67 ± 11.37	523.67 ± 34.08	6.30 ± 0.00	6.38 ± 0.00	1430 ± 1	1450 ± 1	−13.07 ± 0.12	−14.20 ± 0.36
PN 3	21	339.40 ± 9.14	335.77 ± 7.46	0.13 ± 0.02	0.11 ± 0.02	530.67 ± 17.95	513.33 ± 28.71	6.61 ± 0.00	6.73 ± 0.01	1668 ± 3	1675 ± 2	−7.23 ± 0.53	−10.63 ± 0.12
PN 3	22	333.17 ± 6.04	334.60 ± 7.61	0.12 ± 0.03	0.12 ± 0.00	508.00 ± 19.92	512.33 ± 24.95	6.06 ± 0.00	6.05 ± 0.01	1412 ± 3	1400 ± 6	−24.13 ± 0.60	−23.10 ± 0.78
PN 3	23	349.63 ± 7.36	330.80 ± 5.01	0.14 ± 0.01	0.10 ± 0.02	558.33 ± 24.79	494.67 ± 17.01	6.01 ± 0.01	6.02 ± 001	1530 ± 5	1572 ± 6	−17.60 ± 0.56	−20.47 ± 0.42
PN 3	24	323.97 ± 5.59	340.50 ± 18.04	0.10 ± 0.04	0.14 ± 0.02	479.33 ± 16.77	534.00 ± 50.48	6.01 ± 0.00	6.02 ± 0.01	1617 ± 7	1636 ± 7	−21.27 ± 2.10	−16.83 ± 1.66
PN 3	25	344.50 ± 8.31	332.37 ± 1.27	0.14 ± 0.01	0.11 ± 0.04	540.67 ± 21.13	502.00 ± 15.00	6.04 ± 0.00	6.07 ± 0.01	1383 ± 15	1382 ± 5	−20.73 ± 0.38	−20.57 ± 0.15
PN 3	26	332.73 ± 2.40	342.63 ± 17.84	0.10 ± 0.01	0.10 ± 0.04	502.33 ± 7.77	526.33 ± 53.01	6.02 ± 0.01	6.07 ± 0,00	1367 ± 4	1383 ± 4	−18.53 ± 0.31	−15.73 ± 0.12
PN 3	27	337.50 ± 6.95	337.97 ± 11.14	0.12 ± 0.02	0.11 ± 0.03	517.67 ± 12.58	516.00 ± 28.16	6.01 ± 0.00	6.05 ± 0.00	1390 ± 4	1382 ± 3	−14.77 ± 0.49	−14.47 ± 0.38

PN 3	28	376.23 ± 25.72	337.57 ± 5.08	0.20 ± 0.03	0.10 ± 0.01	683.67 ± 22.12	511.33 ± 11.02	6.04 ± 0.00	6.07 ± 0.00	1371 ± 9	1380 ± 1	−21.37 ± 0.29	−22.10 ± 0.75
PN 3	29	344.93 ± 12.00	333.07 ± 8.63	0.12 ± 0.02	0.10 ± 0.02	541.33 ± 42.45	496.67 ± 14.47	6.04 ± 0.01	6.08 ± 0.01	1315 ± 7	1337 ± 3	−9.68 ± 0.41	−10.97 ± 0.40
PN 3	30	348.03 ± 12.62	331.37 ± 6.60	0.18 ± 0.02	0.10 ± 0.02	590.00 ± 37.36	498.33 ± 20.60	6.05 ± 0.00	6.09 ± 0.00	1285 ± 4	1290 ± 5	−5.51 ± 0.10	−7.19 ± 0.30
PN 3	31	339.37 ± 10.40	340.00 ± 1.40	0.11 ± 0.02	0.10 ± 0.00	529.67 ± 33.32	516.33 ± 1.15	6.02 ± 0.00	6.06 ± 0.00	1360 ± 11	1374 ± 4	−16.70 ± 0.10	−18.33 ± 0.42
PN 3	32	353.33 ± 28.27	329.83 ± 7.88	0.17 ± 0.02	0.10 ± 0.03	587.67 ± 71.25	491.00 ± 32.23	6.03 ± 0.01	6.07 ± 0.01	1323 ± 1	1326 ± 2	−8.00 ± 0.21	−9.05 ± 0.74
PN 3	33	329.80 ± 8.81	327.53 ± 2.38	0.10 ± 0.05	0.09 ± 0.03	488.67 ± 26.39	480.33 ± 10.21	6.05 ± 0.00	6.08 ± 0.00	1252 ± 9	1286 ± 10	−3.90 ± 0.34	−5.17 ± 0.03
PN 3	34	336.37 ± 16.96	334.80 ± 4.10	0.20 ± 0.00	0.11 ± 0.01	573.33 ± 31.72	515.33 ± 16.07	6.00 ± 0.01	6.02 ± 0.00	1392 ± 2	1414 ± 1	−15.17 ± 0.31	−15.47 ± 0.15
PN 3	35	337.50 ± 6.02	329.77 ± 8.08	0.11 ± 0.02	0.11 ± 0.02	515.67 ± 15.50	498.67 ± 18.18	6.00 ± 0.00	6.02 ± 0.00	1430 ± 10	1450 ± 11	−7.16 ± 0.20	−8.26 ± 0.39
PN 3	36	367.43 ± 26.14	329.20 ± 2.86	0.23 ± 0.00	0.10 ± 0.02	683.00 ± 66.55	495.00 ± 11.36	6.00 ± 0.01	6.05 ± 0.00	1516 ± 5	1516 ± 2	−3.81 ± 0.12	−4.73 ± 0.68
PN 3	37	333.33 ± 1.11	335.57 ± 5.71	0.10 ± 0.03	0.10 ± 0.01	499.33 ± 13.80	503.33 ± 16.50	6.38 ± 0.01	6.41 ± 0.01	1405 ± 1	1393 ± 2	−29.80 ± 0.35	−31.27 ± 0.71
PN 3	38	339.23 ± 3.01	340.53 ± 8.14	0.11 ± 0.02	0.12 ± 0.01	522.67 ± 14.36	532.67 ± 32.25	6.76 ± 0.00	6.80 ± 0.00	1424 ± 24	1383 ± 14	−33.10 ± 0.00	−35.97 ± 0.15
PN 3	39	341.53 ± 14.50	337.73 ± 8.45	0.11 ± 0.01	0.12 ± 0.02	529.00 ± 42.51	520.00 ± 15.62	7.01 ± 0.00	7.04 ± 0.00	1399 ± 3	1428 ± 9	−32.37 ± 0.93	−32.67 ± 0.71
PN 3	40	337.97 ± 3.21	337.33 ± 2.61	0.11 ± 0.01	0.11 ± 0.01	516.00 ± 6.56	523.00 ± 4.36	6.30 ± 0.01	6.44 ± 0.03	1394 ± 6	1430 ± 11	−16.97 ± 0.31	−17.50 ± 0.62
PN 3	41	348.13 ± 1.86	329.17 ± 5.37	0.14 ± 0.01	0.10 ± 0.03	559.00 ± 5.20	489.00 ± 18.52	6.56 ± 0.00	6.60 ± 0.00	1449 ± 0	1482 ± 1	−10.09 ± 0.62	−10.77 ± 0.42
PN 3	42	330.77 ± 3.27	341.40 ± 11.34	0.12 ± 0.03	0.12 ± 0.05	498.00 ± 11.53	529.33 ± 51.33	6.72 ± 0.00	6.76 ± 0.01	1506 ± 2	1561 ± 2	−7.07 ± 0.41	−8.20 ± 0.37
PN 4	1	335.23 ± 2.63	330.90 ± 6.56	0.12 ± 0.02	0.11 ± 0.01	522.67 ± 16.26	497.33 ± 17.16	6.01 ± 0.00	6.03 ± 0.00	1340 ± 3	1339 ± 16	−27.03 ± 0.76	−32.13 ± 0.57
PN 4	2	333.10 ± 5.03	323.47 ± 2.87	0.13 ± 0.03	0.08 ± 0.02	529.33 ± 26.16	476.33 ± 21.55	5.98 ± 0.00	6.00 ± 0.01	1392 ± 1	1407 ± 5	−14.00 ± 0.82	−21.80 ± 0.53
PN 4	3	331.87 ± 7.52	325.80 ± 7.93	0.10 ± 0.03	0.14 ± 0.02	504.00 ± 32.23	511.33 ± 26.86	5.97 ± 0.00	5.98 ± 0.00	1641 ± 3	1654 ± 1	−16.37 ± 0.38	−14.30 ± 0.46
PN 4	4	326.83 ± 7.81	323.50 ± 2.63	0.11 ± 0.03	0.12 ± 0.03	496.67 ± 28.92	495.00 ± 20.07	6.06 ± 0.00	6.08 ± 0.00	1338 ± 1	1359 ± 5	−33.73 ± 1.50	−29.67 ± 1.02
PN 4	5	335.23 ± 2.63	330.90 ± 6.56	0.12 ± 0.02	0.11 ± 0.01	522.67 ± 16.26	497.33 ± 17.16	5.99 ± 0.00	6.02 ± 0.00	1343 ± 4	1351 ± 2	−21.63 ± 0.40	−15.37 ± 0.75
PN 4	6	332.23 ± 18.15	330.93 ± 5.47	0.11 ± 0.04	0.11 ± 0.02	513.67 ± 63.69	511.67 ± 17.01	5.97 ± 0.00	5.99 ± 0.00	1337 ± 7	1349 ± 5	−13.17 ± 0.60	−11.87 ± 0.55
PN 4	7	329.60 ± 9.13	323.63 ± 1.33	0.11 ± 0.02	0.10 ± 0.03	507.67 ± 32.50	489.33 ± 17.10	6.00 ± 0.00	603 ± 0.01	1341 ± 5	1358 ± 3	−23.83 ± 0.42	−23.27 ± 0.38
PN 4	8	328.03 ± 7.05	326.17 ± 7.98	0.10 ± 0.02	0.10 ± 0.03	499.00 ± 19.08	489.00 ± 36.39	6.00 ± 0.00	6.01 ± 0.00	1329 ± 3	1351 ± 5	−16.30 ± 0.56	−15.83 ± 0.29
PN 4	9	325.70 ± 1.55	332.87 ± 7.30	0.10 ± 0.01	0.13 ± 0.00	490.00 ± 13.23	521.33 ± 17.10	6.00 ± 0.00	6.01 ± 0.00	1300 ± 5	1320 ± 5	−11.10 ± 0.17	−10.73 ± 0.67
PN 4	10	325.93 ± 11.03	330.13 ± 4.94	0.12 ± 0.02	0.12 ± 0.01	495.67 ± 36.25	515.67 ± 17.95	6.00 ± 0.00	6.02 ± 0.00	1331 ± 1	1337 ± 10	−24.00 ± 0.89	−22.93 ± 0.65
PN 4	11	328.30 ± 9.40	333.73 ± 3.36	0.11 ± 0.01	0.12 ± 0.01	498.67 ± 28.73	518.33 ± 8.96	5.98 ± 0.00	6.00 ± 0.00	1314 ± 7	1344 ± 2	−13.33 ± 0.40	−13.77 ± 0.15
PN 4	12	331.63 ± 5.37	336.00 ± 3.83	0.11 ± 0.01	0.14 ± 0.02	512.00 ± 15.10	537.00 ± 13.45	5.99 ± 0.00	6.01 ± 0.00	1301 ± 2	1325 ± 13	-7.21 ± 0.10	-6.84 ± 0.63
PN 4	13	334.73 ± 5.56	328.67 ± 7.91	0.12 ± 0.01	0.12 ± 0.02	521.00 ± 1.,06	507.33 ± 11.37	5.98 ± 0.00	6.01 ± 0.00	1348 ± 6	1369 ± 4	−21.47 ± 0.90	−20.40 ± 0.26
PN 4	14	330.60 ± 5.65	330.00 ± 4.26	0.12 ± 0.03	0.10 ± 0.01	510.00 ± 14.93	502.00 ± 12.12	5.95 ± 0.01	5.98 ± 0.01	1371 ± 6	1413 ± 5	−12.43 ± 0.64	−12.67 ± 0.12
PN 4	15	330.97 ± 9.25	338.73 ± 7.48	0.10 ± 0.02	0.13 ± 0.01	505.67 ± 26.08	541.00 ± 23.64	5.98 ± 0.01	5.98 ± 0.00	1615 ± 9	1633 ± 3	−6.13 ± 0.37	−6.30 ± 0.41
PN 4	16	327.47 ± 7.96	330.70 ± 13.20	0.11 ± 0.02	0.15 ± 0.01	492.67 ± 31.07	521.33 ± 30.50	6.25 ± 0.00	6.31 ± 0.00	1347 ± 3	1374 ± 7	−27.60 ± 0.36	−25.23 ± 0.67

PN 4	17	325.60 ± 3.47	328.43 ± 6.54	0.10 ± 0.02	0.11 ± 0.00	490.67 ± 23.63	506.00 ± 9.54	6.45 ± 0.00	6.48 ± 0.00	1339 ± 4	1383 ± 10	−30.20 ± 0.98	−36.03 ± 0.40
PN 4	18	332.63 ± 6.46	331.93 ± 7.42	0.13 ± 0.02	0.12 ± 0.02	515.33 ± 15.50	520.67 ± 35.50	7.01 ± 0.00	7.03 ± 0.00	1359 ± 0	1388 ± 5	−31.23 ± 0.74	−31.83 ± 0.68
PN 4	19	328.43 ± 2.67	323.23 ± 0.68	0.11 ± 0.00	0.13 ± 0.01	503..33 ± 3.79	501.33 ± 7.57	6.20 ± 0.00	6.29 ± 0.00	1343 ± 1	1365 ± 6	−22.90 ± 0.26	−24.77 ± 0.70
PN 4	20	333.30 ± 6.80	337.43 ± 10.20	0.10 ± 0.01	0.14 ± 0.02	508.33 ± 17.90	548.67 ± 32.25	6.31 ± 0.00	6.36 ± 0.01	1382 ± 5	1400 ± 2	−14.07 ± 0.31	−14.60 ± 0.72
PN 4	21	335.70 ± 8.45	323.60 ± 3.21	0.12 ± 0.01	0.09 ± 0.02	524.00 ± 21.66	482.67 ± 22.48	6.71 ± 0.01	6.74 ± 0.00	1629 ± 1	1647 ± 10	−9.11 ± 0.71	−8.98 ± 0.47
PN 4	22	323.83 ± 9.53	326.03 ± 6.44	0.10 ± 0.02	0.11 ± 0.02	494.67 ± 25.97	500.33 ± 28.15	6.03 ± 0.03	6.03 ± 0.00	1379 ± 18	1407 ± 21	−28.63 ± 1.00	−30.73 ± 1.18
PN 4	23	325.43 ± 2.67	330.10 ± 3.30	0.11 ± 0.00	0.11 ± 0.02	504.33 ± 9.02	512.67 ± 11.68	5.99 ± 0.01	6.02 ± 0.00	1466 ± 10	1469 ± 3	−22.00 ± 0.36	−21.87 ± 0.50
PN 4	24	321.23 ± 3.50	320.80 ± 2.88	0.09 ± 0.04	0.10 ± 0.00	488.67 ± 19.30	489.00 ± 6.08	6.00 ± 0.01	6.02 ± 0.01	1584 ± 1	1599 ± 2	−18.53 ± 1.34	−19.87 ± 2.23
PN 4	25	315.67 ± 14.08	328.47 ± 13.71	0.13 ± 0.01	0.11 ± 0.03	478.00 ± 37.36	506.67 ± 45.54	6.05 ± 0.01	6.06 ± 0.01	1349 ± 0	1356 ± 3	−24.93 ± 0.45	−22.90 ± 0.36
PN 4	26	324.43 ± 5.82	320.23 ± 13.04	0.13 ± 0.01	0.12 ± 0.04	508.33 ± 16.26	492.67 ± 42.90	6.04 ± 0.00	6.05 ± 0.00	1338 ± 7	1366 ± 5	−16.33 ± 0.25	−20.60 ± 0.26
PN 4	27	329.90 ± 9.42	320.30 ± 5.55	0.13 ± 0.01	0.10 ± 0.00	518.67 ± 18.23	485.67 ± 17.50	6.02 ± 0.00	6.04 ± 0.00	1334 ± 4	1362 ± 14	−13.87 ± 0.76	−14.43 ± 0.42
PN 4	28	333.67 ± 8.27	334.77 ± 8.58	0.12 ± 0.02	0.13 ± 0.01	521.00 ± 21.00	541.67 ± 27.75	6.05 ± 0.00	6.07 ± 0.01	1334 ± 6	1374 ± 1	−23.13 ± 0.42	−23.10 ± 0.66
PN 4	29	323.67 ± 4.17	329.07 ± 13.94	0.11 ± 0.00	0.11 ± 0.03	494.67 ± 11.06	505.33 ± 46.76	6.04 ± 0.01	6.07 ± 0.00	1314 ± 1	1316 ± 9	−11.40 ± 0.20	−12.60 ± 0.10
PN 4	30	326.33 ± 11.44	321.50 ± 3.15	0.10 ± 0.02	0.10 ± 0.01	495.67 ± 24.01	485.00 ± 9.00	6.05 ± 0.00	6.08 ± 0.00	1259 ± 5	1289 ± 6	−7.52 ± 0.45	−6.66 ± 0.24
PN 4	31	321.37 ± 7.81	311.53 ± 9.00	0.12 ± 0.02	0.11 ± 0.03	490.67 ± 22.03	456.67 ± 9.45	6.03 ± 0.00	6.05 ± 0.00	1338 ± 2	1365 ± 16	−17.10 ± 0.40	−17.57 ± 0.42
PN 4	32	325.23 ± 8.95	320.13 ± 5.46	0.12 ± 0.02	0.12 ± 0.01	507.33 ± 20.13	484.33 ± 18.77	6.04 ± 0.01	6.06 ± 0.01	1314 ± 6	1310 ± 0	−8.72 ± 0.19	−8.80 ± 0.11
PN 4	33	323.17 ± 5.58	321.60 ± 9.51	0.11 ± 0.01	0.10 ± 0.03	490.33 ± 14.74	484.67 ± 27.01	6.06 ± 0..01	6.07 ± 0.00	1256 ± 7	1276 ± 2	−4.62 ± 0.21	−4.77 ± 0.30
PN 4	34	319.07 ± 3.78	320.03 ± 9.02	0.10 ± 0.01	0.10 ± 0.01	490.67 ± 17.56	480.67 ± 24.70	6.00 ± 0.00	6.02 ± 0.01	1367 ± 0	1410 ± 1	−16.17 ± 0.12	−15.33 ± 0.32
PN 4	35	322.73 ± 10.51	322.57 ± 7.46	0.13 ± 0.02	0.11 ± 0.02	492.33 ± 31.01	493.00 ± 27.73	6.00 ± 0.00	6.03 ± 0.01	1412 ± 2	1455 ± 17	−7.59 ± 0.34	−8.49 ± 0.04
PN 4	36	315.50 ± 4.85	326.60 ± 11.70	0.07 ± 0.03	0.11 ± 0.01	464.33 ± 24.03	510.67 ± 30.44	6.02 ± 0.00	6.04 ± 0.00	1485 ± 3	1508 ± 11	−4.83 ± 0.38	−4.35 ± 0.31
PN 4	37	322.80 ± 8.76	321.10 ± 10.51	0.11 ± 0.02	0.11 ± 0.02	492.33 ± 28.59	486.33 ± 30.17	6.38 ± 0.00	6.41 ± 0.00	1349 ± 6	1385 ± 4	−26.67 ± 0.74	−27.73 ± 0.60
PN 4	38	322.20 ± 8.26	319.23 ± 3.07	0.11 ± 0.02	0.10 ± 0.02	492.33 ± 32.59	484.00 ± 23.26	6.78 ± 0.00	6.80 ± 0.01	1358 ± 2	1384 ± 16	−40.83 ± 0.40	−34.87 ± 0.55
PN 4	39	323.37 ± 6.95	324.07 ± 12.46	0.12 ± 0.01	0.11 ± 0.03	502.67 ± 16.07	498.00 ± 39.40	7.04 ± 0.00	7.05 ± 0.00	1374 ± 1	1393 ± 1	−34.50 ± 0.75	−36.03 ± 0.58
PN 4	40	318.47 ± 10.68	327.27 ± 14.71	0.12 ± 0.01	0.11 ± 0.02	481.33 ± 25.32	503.67 ± 50.56	6.38 ± 0.01	6.44 ± 0.01	1377 ± 2	1385 ± 4	−18.37 ± 0.29	−19.73 ± 0.83
PN 4	41	327.73 ± 4.79	322.47 ± 2.11	0.13 ± 0.02	0.11 ± 0.03	506.67 ± 9.50	492.67 ± 764	6.58 ± 0.00	6.61 ± 0.01	1437 ± 3	1449 ± 2	−11.77 ± 0.61	−11.77 ± 0.74
PN 4	42	321.60 ± 3.39	322.93 ± 3.98	0.11 ± 0.01	0.10 ± 0.00	489.00 ± 13.11	489.33 ± 0.58	6.75 ± 0.01	6.78 ± 0.01	1520 ± 4	1552 ± 10	−7.70 ± 0.46	−8.24 ± 0.16

MDD – mean droplet diameter obtained by diffraction light scattering method
Dv(90) – 90% of the particles lie below the value
